# Efficient Hardware Implementation of the Lightweight Block Encryption Algorithm LEA

**DOI:** 10.3390/s140100975

**Published:** 2014-01-08

**Authors:** Donggeon Lee, Dong-Chan Kim, Daesung Kwon, Howon Kim

**Affiliations:** 1 Department of Computer Engineering, Pusan National University, Busan 609-735, Korea; E-Mail: howonkim@pusan.ac.kr; 2 Attached Institute of Electronic and Telecommunication Research Institute, Daejeon 305-390, Korea; E-Mails: dongchan@ensec.re.kr (D.-C.K.); ds_kwon@ensec.re.kr (D.K.)

**Keywords:** LEA, lightweight block cipher, hardware implementation, FPGA, ASIC

## Abstract

Recently, due to the advent of resource-constrained trends, such as smartphones and smart devices, the computing environment is changing. Because our daily life is deeply intertwined with ubiquitous networks, the importance of security is growing. A lightweight encryption algorithm is essential for secure communication between these kinds of resource-constrained devices, and many researchers have been investigating this field. Recently, a lightweight block cipher called LEA was proposed. LEA was originally targeted for efficient implementation on microprocessors, as it is fast when implemented in software and furthermore, it has a small memory footprint. To reflect on recent technology, all required calculations utilize 32-bit wide operations. In addition, the algorithm is comprised of not complex S-Box-like structures but simple Addition, Rotation, and XOR operations. To the best of our knowledge, this paper is the first report on a comprehensive hardware implementation of LEA. We present various hardware structures and their implementation results according to key sizes. Even though LEA was originally targeted at software efficiency, it also shows high efficiency when implemented as hardware.

## 1. Introduction

Recent improvements in semi-conductor technology have enabled the computing environment to become mobile, and accelerated the change to a ubiquitous era. The use of small mobile devices is growing explosively, and the importance of security is increasing daily. One of the essential ingredients of smart device security is a block cipher, and lightweight energy-efficient implementation techniques are required for small mobile devices.

Techniques for securing resource-constrained devices such as RFID (Radio-frequency Identification) tags have been proposed. In 2005, Lim and Korkishko [1] presented a lightweight block cipher called mCrypton that encrypts plaintext into ciphertext by using 4 by 4 nibble (4-bit) matrix-based simple operations such as substitution (S-Box), permutation, transposition, and key addition (XOR). The following year, Hong *et al*. [2] proposed a lightweight block cipher called HIGHT, which has a Feistel structure and operates with simple calculations such as XOR, addition, subtraction, and rotation. In 2007, Bogdanov *et al*. [3] introduced PRESENT, which is comprised of substitution, permutation, and XOR. In 2009, KATAN and KTANTAN were proposed by Cammoere *et al*. [4] KATAN divides plaintext into two parts and stores them into two registers, and the outputs from non-linear functions are stored in the least significant bit (LSB) of each other's register. On the other hand KTANTAN is a fixed-key version of KATAN and has a different key scheduling scheme. In the same year, Rotor-based Humming Bird was proposed by Revere Security. However, these algorithms have been revealed to be vulnerable to chosen-IV attacks and chosen message attacks. Two years later, HummingBird2 [5], an improved version of HummingBird, was proposed. In 2011, Guo *et al*. [6] proposed a lightweight cipher LED, with a structure similar to AES, but it does not perform key scheduling.

Both lightweight block ciphers and methods to optimize legacy block ciphers have been studied. Moradi *et al*. [7] optimized AES and reduced the gate count to 2,400 GE (gate equivalent). Poschmann *et al*. [8] implemented DES with 1,848 GE.

Recently, the Electronics and Telecommunications Research Institute in Korea announced a new lightweight block cipher called LEA [9]. The focus of LEA design is a “software-oriented lightweightness” for resource-constrained small devices. It is intended to have a small code size and consume low power. Therefore, it is extremely efficient when it is implemented in software. LEA has three key sizes of 128, 192, or 256 bits and a 128-bit block size. Every inner operation of the LEA is 32 bits wide, since 32-bit microprocessors are more popular than 8-bit ones these days. Further, it does not employ a complex operation such as S-Box, and only uses simple operations such as addition, rotation, and XOR (ARX).

Usually, small chip size and reasonably fast encryption is preferred for cryptographic hardware for small devices in resource constrained environments such as RFID tags or smart meters for smart grids. In this paper, we propose several methods to optimize LEA hardware for all key sizes and present implementation results in terms of time and chip area cost. This work is the first that studies a comprehensive hardware implementation of LEA. LEA was originally designed for software implementation, but we aim to demonstrate that it is also efficient when implemented in hardware.

The rest of this paper is organized as follows: We introduce the LEA algorithm in Section 2, and then present elemental techniques for implementing LEA in hardware in Section 3. Section 4 presents hardware structures for the 128, 192, and 256 key version of LEA, and corresponding implementation results are presented in Section 5. We conclude this paper in Section 6.

## 2. LEA Algorithm

In this section, we introduce the LEA block cipher. LEA has 128 bit long message blocks and 128, 192, or 256 bit long keys. We denote each version of this algorithm as LEA-128, LEA-196, and LEA-256 according to key length.

### 2.1. Notations

We present notations and corresponding descriptions required to explain the LEA algorithm in Table 1.

### 2.2. Key Schedule

#### 2.2.1. Constants

4, 6, and 8 constant values that are 32 bits long are used for each version of the LEA key schedule. Each constant is defined as follows:

(1) $$\begin{array}{ll} \delta_{0} & =C3EFE9DB_{16},\delta_{1}=44626B02_{16}\\ \delta_{2} & =79E27C8A_{16},\delta_{3}=78DF30EC_{16}\\ \delta_{4} & =715EA49E_{16},\delta_{5}=C785DA0A_{16}\\ \delta_{6} & =E04EF22A_{16},\delta_{7}=E5C40957_{16} \end{array}$$

The constants are generated from the hexadecimal expression of $\sqrt{766,995}$, where 76, 69, and 95 are ASCII codes for “L”, “E”, and “A”.

#### 2.2.2. Key Schedule for 128-Bit Key

At the beginning of the LEA-128 key schedule, the key state *T* is assigned as $T_{n}^{-1}=K_{n}$ where 0 ≤ *n* < 4. The key schedule of LEA-128 is defined as follows:

(2) $$\begin{array}{l} T_{0}^{i+1}\leftarrow ROL_{1}(T_{0}^{i}⊞ROL_{i}({\delta_{i}\;}_{\mathrm{mod}\;4}))\\ T_{1}^{i+1}\leftarrow ROL_{3}(T_{1}^{i}⊞ROL_{i+1}({\delta_{i}\;}_{\mathrm{mod}\;4}))\\ T_{2}^{i+1}\leftarrow ROL_{6}(T_{2}^{i}⊞ROL_{i+2}({\delta_{i}\;}_{\mathrm{mod}\;4}))\\ T_{3}^{i+1}\leftarrow ROL_{11}(T_{3}^{i}⊞ROL_{i+3}({\delta_{i}\;}_{\mathrm{mod}\;4}))\\ RK^{i}\leftarrow (T_{0}^{i},T_{1}^{i},T_{2}^{i},T_{1}^{i},T_{3}^{i},T_{1}^{i}) \end{array}$$

#### 2.2.3. Key Schedule for 192-Bit Key

The key schedule of LEA-192 also starts with setting *T* as $T_{n}^{-1}=K_{n}$ where 0 ≤ *n* < 6. The key schedule of LEA-192 is defined as follows:

(3) $$\begin{array}{l} T_{0}^{i+1}\leftarrow ROL_{1}(T_{0}^{i}⊞ROL_{i}({\delta_{i}\;}_{\mathrm{mod}\;6}))\\ T_{1}^{i+1}\leftarrow ROL_{3}(T_{1}^{i}⊞ROL_{i+1}({\delta_{i}\;}_{\mathrm{mod}\;6}))\\ T_{2}^{i+1}\leftarrow ROL_{6}(T_{2}^{i}⊞ROL_{i+2}({\delta_{i}\;}_{\mathrm{mod}\;6}))\\ T_{3}^{i+1}\leftarrow ROL_{11}(T_{3}^{i}⊞ROL_{i+3}({\delta_{i}\;}_{\mathrm{mod}\;6}))\\ T_{4}^{i+1}\leftarrow ROL_{13}(T_{4}^{i}⊞ROL_{i+4}({\delta_{i}\;}_{\mathrm{mod}\;6}))\\ T_{5}^{i+1}\leftarrow ROL_{17}(T_{5}^{i}⊞ROL_{i+5}({\delta_{i}\;}_{\mathrm{mod}\;6}))\\ RK^{i}\leftarrow (T_{0}^{i},T_{1}^{i},T_{2}^{i},T_{3}^{i},T_{4}^{i},T_{5}^{i}) \end{array}$$

#### 2.2.4. Key Schedule for 256-Bit Key

Likewise, the key schedule of LEA-256 starts with setting *T* as $T_{n}^{-1}=K_{n}$ where 0 ≤ *n* < 8, and is defined as follows:

(4) $$\begin{array}{l} {T_{6i}^{i+1\;}}_{\mathrm{mod}\;8}\leftarrow ROL_{1}({T_{6i}^{i}}_{\mathrm{mod}8}⊞ROL_{i}({\delta_{i}\;}_{\mathrm{mod}\;8}))\\ {T_{6i+1}^{i+1\;}}_{\mathrm{mod}\;8}\leftarrow ROL_{3}({T_{6i+1}^{i}\;}_{\mathrm{mod}8}⊞ROL_{i+1}({\delta_{i}\;}_{\mathrm{mod}\;8}))\\ {T_{6i+2}^{i+1\;}}_{\mathrm{mod}\;8}\leftarrow ROL_{6}({T_{6i+2}^{i}}_{\mathrm{mod}8}⊞ROL_{i+2}({\delta_{i}\;}_{\mathrm{mod}\;8}))\\ {T_{6i+3}^{i+1\;}}_{\mathrm{mod}\;8}\leftarrow ROL_{11}({T_{6i+3}^{i}}_{\mathrm{mod}8}⊞ROL_{i+3}({\delta_{i}\;}_{\mathrm{mod}\;8}))\\ {T_{6i+4}^{i+1\;}}_{\mathrm{mod}\;8}\leftarrow ROL_{13}({T_{6i+4}^{i}}_{\mathrm{mod}8}⊞ROL_{i+4}({\delta_{i}\;}_{\mathrm{mod}\;8}))\\ {T_{6i+5}^{i+1\;}}_{\mathrm{mod}\;8}\leftarrow ROL_{17}({T_{6i+5}^{i}}_{\mathrm{mod}8}⊞ROL_{i+5}({\delta_{i}\;}_{\mathrm{mod}\;8}))\\ RK^{i}\leftarrow (T_{0}^{i},T_{1}^{i},T_{2}^{i},T_{3}^{i},T_{4}^{i},T_{5}^{i}) \end{array}$$

### 2.3. Encryption Procedure

**Figure 1.**
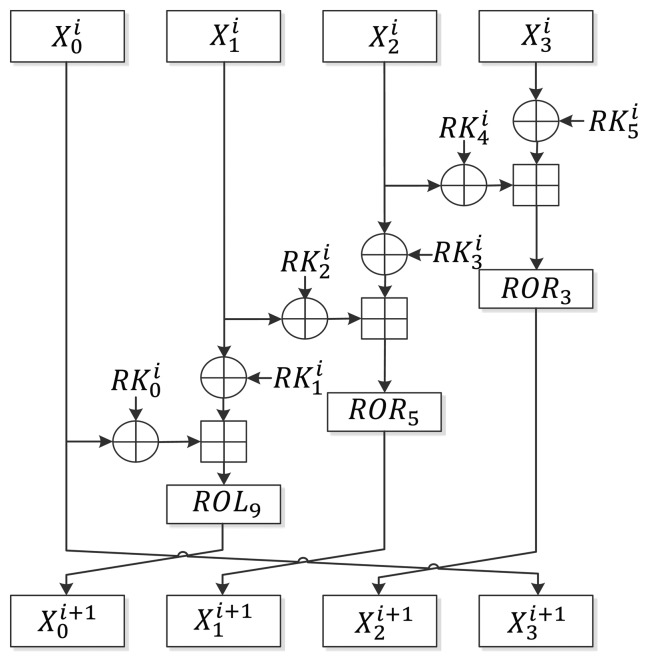
Round function of LEA.

As described in Section 2.1, LEA-128/192/256 iterates in 24/28/32 rounds. Unlike AES [10] or HIGHT [2], which require a special final round function, LEA uses only one round function. Figure 1 shows the round function of LEA. At the beginning of the encryption, the intermediate state *X* is set as $X_{n}^{0}=P_{n}$ where 0 ≤ *n* < 4 and the following round function is executed *r* times:

(5) $$\begin{array}{l} X_{0}^{i+1}\leftarrow ROL_{9}((X_{0}^{i}⊕RK_{0}^{i})⊞(X_{1}^{i}⊕RK_{1}^{i}))\\ X_{1}^{i+1}\leftarrow ROR_{5}((X_{1}^{i}⊕RK_{2}^{i})⊞(X_{2}^{i}⊕RK_{3}^{i}))\\ X_{2}^{i+1}\leftarrow ROR_{3}((X_{2}^{i}⊕RK_{4}^{i})⊞(X_{3}^{i}⊕RK_{5}^{i}))\\ X_{3}^{i+1}\leftarrow X_{0}^{i} \end{array}$$

The final $C_{n}=X_{n}^{r}$ is generated and used as ciphertext where 0 ≤ *n* < 4.

## 3. Elemental Hardware Structures for LEA Calculation

This section describes elemental hardware structures used for implementing LEA hardware.

### 3.1. Constant Value Schedule Logic for Speed-Optimized Implementation

LEA employs several constants for key scheduling. To design the constant schedule logic, the usage patterns of constants need to be analyzed. In Equation (5), the constant values used for the *i*-th round function are *ROL_i_*(*δ_i_*_mod 4_), *ROL_i_*_+1_(*δ_i_*_mod 4_), *ROL_i_*_+2_(*δ_i_*_mod 4_), and *ROL_i_*_+3_(*δ_i_*_mod 4_). At the *i*-th round, the *i* mod 4-th constant is chosen; in other words, constants are used in increasing order, *i.e*., *δ*_0_, *δ*_1_, *δ*_2_, *δ*_3_,*δ*_0_,…. After a constant is chosen, it is rotated *i*,*i* + 1, *i* + 2, and *i* + 3 times to the left.

**Figure 2.**
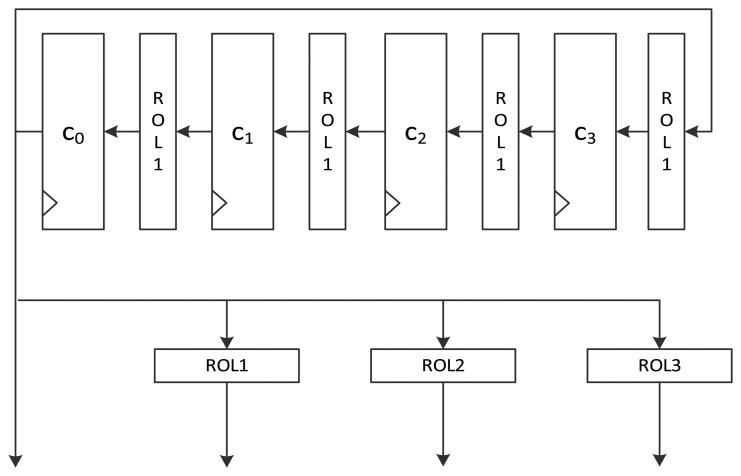
Constant scheduling logic structure for speed-optimized LEA hardware.

Figure 2 shows the intuitive structure of the constant schedule logic of the 128-bit speed-optimized version of LEA hardware. The speed-optimized version executes one round per clock cycle. Therefore, it should generate all four constants required for a round. Constants *δ*_0_ to *δ*_3_ are stored in 32-bit flip-flops *c*_0_ to *c*_3_. Each value in a 32-bit flip-flop moves to the next flip-flop per round. Since a constant value that is rotated *i*-times (*i* + 1, *i* + 2, and *i* + 3 times) is used for the *i*-th round, it is rotated 1 bit left for every round. Since the constant used for the *i*-th round is located at the *c*_0_ register, its value is exactly *ROL_i_*(*δ_i_*_mod 4_). The remaining *ROL_i_*_+1_(*δ_i_*_mod 4_), *ROL_i_*_+2_(*δ_i_*_mod 4_), and *ROL_i_*_+3_(*δ_i_*_mod4_) are generated from corresponding *ROL*_1_, *ROL*_2_, and *ROL*_3_ operations. In the figure, no rotation consumes any logical gates because they can be easily implemented by crossing some wires. Thus, the logic requires only 128 flip-flops.

### 3.2. Constant Value Schedule Logic for Area-Optimized Implementation

**Figure 3.**
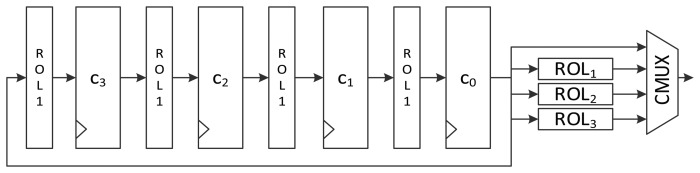
Intuitive constant scheduling logic structure for area-optimized LEA hardware.

To minimize the number of gates required, some logic gates are shared and iteratively used in a round. In area-optimized implementation, one round can be split into several clock cycles. Therefore, four constants must be generated one by one in a round. The intuitive structure of constant scheduling logic is depicted in Figure 3. At the beginning of a round, *c*_0_ is fed with *ROL_i_*(*δ_i_*_mod 4_) from *c*_1_. The value is passed to the key scheduling logic through the first path of the MUX. For the remaining clock cycles of one round, *ROL_i_*_+1_(*δ_i_*_mod 4_), *ROL_i_*_+_*_2_*(*δ_i_*_mod 4_), and *ROL_i_*_+3_(*δ_i_*_mod 4_) are fed to the key scheduling logic using the second, third, and fourth path of the MUX.

**Figure 4.**
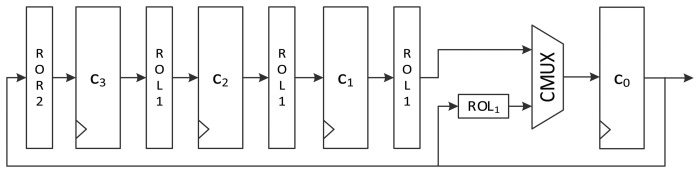
Alternative constant scheduling logic structure for area-optimized LEA hardware.

An alternative logic structure for area-optimized LEA is depicted in Figure 4. The 32-bit constant in *c*_0_ is fed to the key scheduling logic. When the round counter is increased, the upper path of MUX is used, which leads *ROL_i_*(*δ_i_*_mod 4_) at *c*_1_ to move to the *c*_0_ register. In a round, the remaining constant values used for the *i-th* round function, *ROL_i_*_+1_(*δ_i_*_mod 4_), *ROL_i_*_+2_(*δ_i_*_mod 4_), and *ROL_i_*_+3_(*δ_i_*_mod 4_), are generated during the remaining three clock cycles using the lower path of MUX. By using this structure, the cost for the four-input MUX is reduced to that of a two-input MUX. Moreover, the rotating logic before *c*_3_ is different from that in Figure 3. At the final state of a round, the *c*_0_ is *ROL_i_* + 3(*delta_i_*_mod 4_). To make *ROL_i_* + 4(*delta_i_*_mod 4_) have the same value at a register after four rounds, *c*_0_ should be rotated to the right twice. Consequently, the rotation logic before the *c*_3_ register in Figure 3 is different from that in Figure 4.

## 4. Proposed Hardware Structure of LEA

In this section, we describe hardware implementation methods according to three key sizes and the optimization goal(speed or area). Even though the three key versions of LEA use the same round-function, their key scheduling algorithms are different. Therefore, it is impossible to carry out different hardware implementations using the same logic for key scheduling, since they have different structures. The following subsections describe each LEA implementation focused on the key scheduling method. To specify each version according to the key size and optimization goal, each version will be denoted as LEA-KEYSIZE-OPTIMIZATION GOAL (e.g., LEA-128-SPEED refers to the 128-bit version of the LEA implementation with the target of speed improvement).

### 4.1. LEA Implementation Using 128-Bit Key

#### 4.1.1. LEA-128-AREA-1

**Figure 5.**
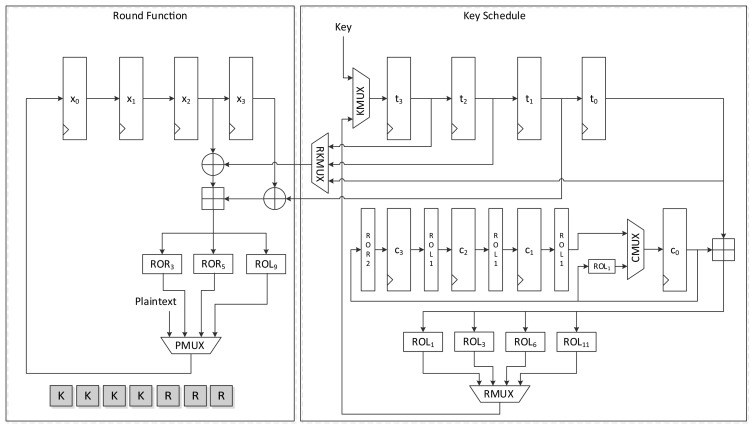
Datapath of LEA-128-AREA-1.

Figure 5 shows the data path of LEA-128-AREA-1. The left side of the data path deals with the round function and the right deals with the scheduling. Twelve 32-bit registers are used. *x*_0_ to *x*_3_ are registers that save the internal state, while *t*_0_ to *t*_3_ are key registers. The remaining four registers, *c*_0_ to *c*_3_, are constant registers.

Plaintexts *X*_0_ to *X*_3_ are supplied to registers *x*_0_ to *x*_3_ in reverse order through the leftmost path of PMUX, and keys *T*_0_ to *T*_3_ are shifted using the upper path of KMUX and stored in registers *t*_0_ to *t*_3_. Four clocks are required to schedule keys, and three clocks are required to update states in a round. Keys in each 32-bit register are scheduled one by one. In accordance with Equation (2), the key in register *t*_0_ is added to a constant and rotated left to a specified number, and is then stored in register *t*_3_. After four clocks of the key scheduling cycle, the round function begins to run. According to Equation (5), two XOR and one addition operations are repeated in a round. For the area-optimized version, we tried to reduce the area by sharing the operations. (*X*_2_, *X*_3_), (*X*_1_, *X*_2_), and (*X*_0_, *X*_1_) are sequentially fed to the two XORs, and both results are added. Scheduled round keys are supplied from registers *t*_0_ to *t*_3_. Since *T*_1_ is always required for the input of one XOR, the output of *t*_1_ is directly connected to the input of the other XOR. The remaining outputs of *t*_0_, *t*_2_, and *t*_3_ are selected by RKMUX, and then keys are supplied in (*RK*_0_, *RK*_1_), (*RK*_2_,*RK*_1_) and (*RK*3,*RK*1) order. The output of the adder is then fed to three rotation logics, and one of them is chosen along with clock cycles and stored in register *x*_0_. In this case, 7 clock cycles are required for a round, thereby completing encryption in 168 clock cycles excluding cycles for input and output.

#### 4.1.2. LEA-128-AREA-2

**Figure 6.**
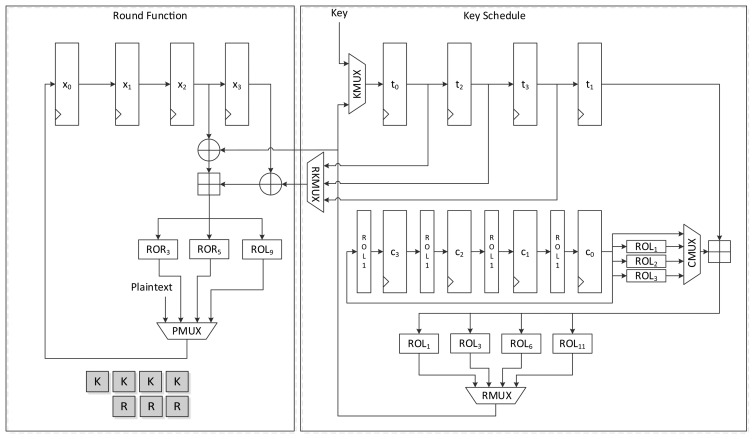
Datapath of LEA-128-AREA-2.

Figure 6 presents another version of the size-optimized LEA-128 implementation. This version reduces the required clock cycles from seven to four compared to LEA-128-AREA-1. The most significant difference between this version and the previous one is that it supplies the schedule key *RK* on the fly. To achieve this, keys are inserted into the register in the order of *T*_1_, *T*_3_, *T*_2_, and *T*_1_. Since *RK*_1_ is always used during a round, it is preferentially scheduled and stored in the *t*_0_ register. Next, *T*_3_ in the *t*_1_ register is scheduled, and the value from RMUX is directly supplied to the XOR operation of the round function. In this way, the remaining keys are also scheduled and used for the round function. Since *RK*_1_ has been moved to registers *t*_0_, *t*_2_, and *t*_3_ along with clock cycles, RKMUX is used to select the register that has *RK*_1_. Since keys are not scheduled in increasing order as in LEA-128-AREA-1, the constant generating logic in Figure 4 cannot be used. Therefore, the logic in Figure 3 is used. In this implementation, one round of operations is carried out in 4 clock cycles, and altogether 96 cycles are required for encryption.

#### 4.1.3. LEA-128-SPEED

**Figure 7.**
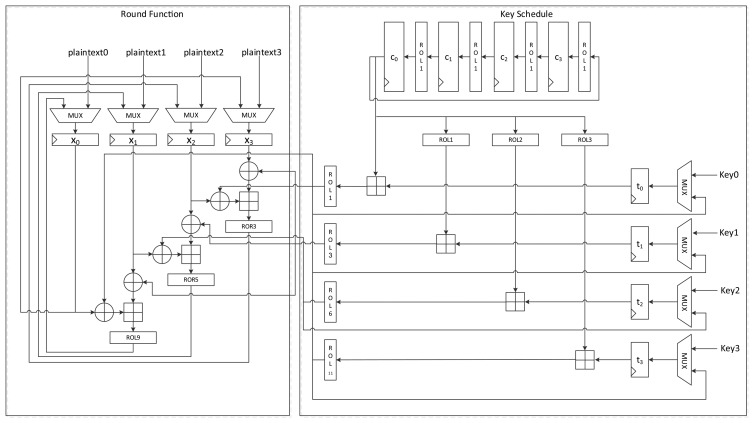
Datapath of LEA-128-SPEED.

Figure 7 shows the data path of LEA-128-SPEED. As seen in the figure, all the required operation logics for a round are arranged for parallel processing in order to execute a round in a clock cycle. Plaintext registers have a MUX for selecting input from an outside or internal state. Further, key registers have a MUX for choosing a key from outside or among the scheduled keys. The constant generation logic in Figure 2 is used.

#### 4.1.4. LEA-192-AREA-1

**Figure 8.**
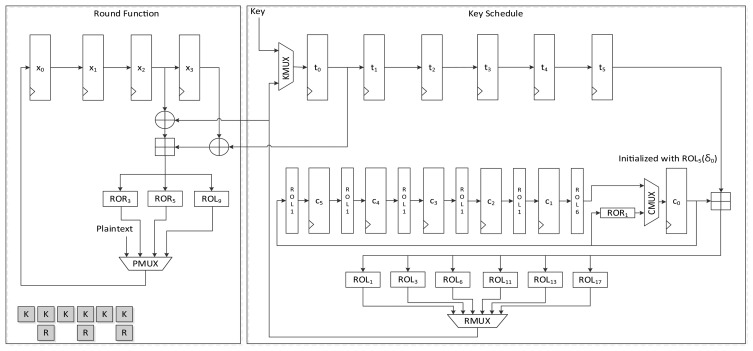
Datapath of LEA-192-AREA-1.

Figure 8 presents the data path of LEA-192-AREA-1. In the case of the 192-bit version of LEA, six 32-bit keys are supplied and six 32-bit constants are used. Unlike LEA-128 which uses *T*_1_ iteratively, LEA-192 uses round keys *T*_0_ to *T*_5_ once in a round. Therefore, a simpler implementation than LEA-128 is possible. This implementation encrypts 128-bit plaintext in 24 clock cycles.

The round function is the same as that used by LEA-128, but it differs in terms of the key schedule logic. First, the key input sequence differs from that found in LEA-128-AREA-1. Keys *T*_5_ to *T*_0_ are scheduled one by one. According to Equation (3), two round keys are used for a round function step. To use the scheduled key on the fly, one of the keys is scheduled in advance and stored in a *t*_0_ register, and is then used for the input of one XOR of the round function. Next, the other key is scheduled and supplied to the other XOR. Since (*X*_2_, *X*_3_) is used first for calculation, (*T*_5_, *T*_4_) should be supplied first. This also changes the constant generation logic. Since constants are used in *ROL_i_*_+5_(*δ_i_*_mod 6_) to *ROL_i_*(*δ_i_*_mod 6_) order, *ROL*_6_(*c*_1_) is moved to the *c*_1_ register at the start of a round. The value is then rotated to the right in every clock. Therefore, *ROL*_1_(*c*_0_) is moved to *c*_5_ when the value in *c*_0_ moves to *c*_5_ at the beginning of a round. Further, register *c*_0_ is initialized with *ROL*_5_(*δ_i_*) for the above reason. This processes one round in 6 clock cycles, and thus 168 clock cycles are required to encrypt a 128-bit message.

#### 4.1.5. LEA-192-AREA-2

**Figure 9.**
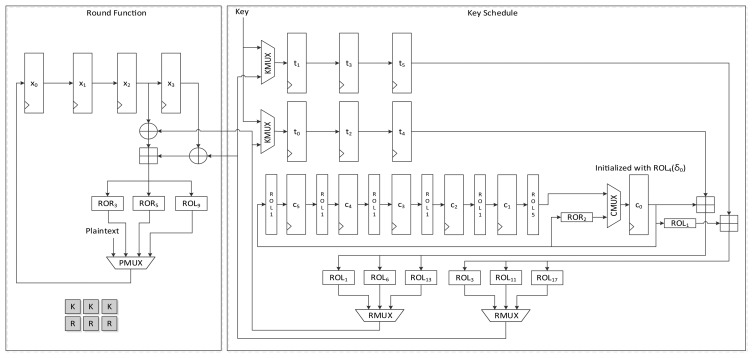
Datapath of LEA-192-AREA-2.

Figure 9 shows the data path of LEA-192-AREA-2, which is a faster version of LEA-192-AREA-1. This implementation schedules two keys in a clock cycle. The sequence of the key input is the same as that used by LEA-192-AREA-1. However, there is a small difference in their constant generation logic. To generate two constants simultaneously, the rotation logic is attached to the *c*_0_ register. Further, one more adder is added. KMUX is divided into two MUXes. The generated round keys are directly supplied to two XORs in the round function. In this implementation, three clock cycles are needed to process a round, and thus 84 clock cycles are needed to encrypt a plaintext block.

#### 4.1.6. LEA-192-SPEED

**Figure 10.**
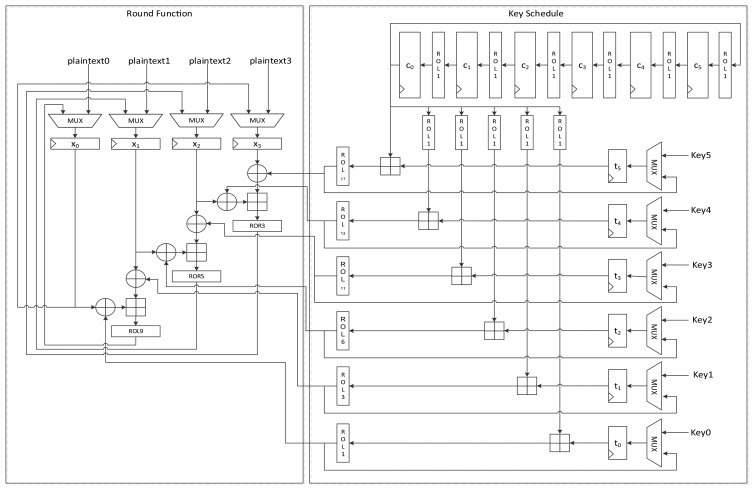
Datapath of LEA-192-SPEED.

LEA-192-SPEED in Figure 10 has the same structure as LEA-128-SPEED, except that it has more registers for keys and constants. It requires 28 clock cycles to encrypt a plaintext block.

#### 4.1.7. LEA-256-AREA-1

**Figure 11.**
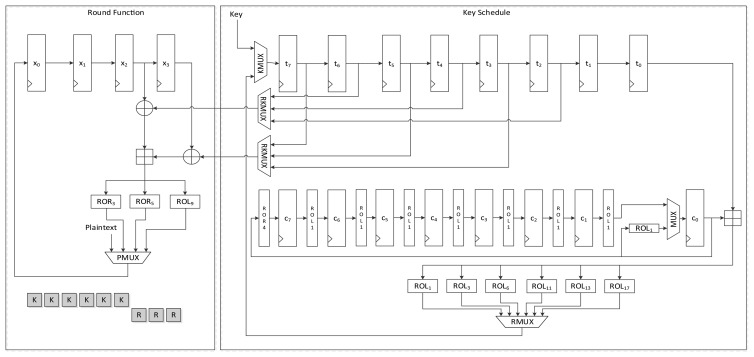
Datapath of LEA-256-AREA-1.

Figure 11 presents the structure of LEA-256-AREA-1. As seen in the area-opt hardware structure of both LEA-128 and LEA-192, they use same hardware structure for the round function. In the implementation, (*X*_2_, *X*_3_), (*X*_1_, *X*_2_), and (*X*_0_, *X*_1_) order, the plaintext(state) blocks are fed to shared operation logic. If this order is changed or reversed, the structure may be complex. For that reason we also used this structure for LEA-256. In this case, the round keys are fed to the operation logic in (*RK*_4_,*RK*_5_), (*RK*_2_, *RK*_3_) and (*RK*_0_, *RK*_1_) order. However, from the Equation (4), key scheduling for LEA-256 in Figure 11 may be the simplest way. This structure schedules keys in *T*_0_ to *T*_5_ order, then the next key generation is started from *T*_6_ and finished at *T*_3_. Therefore, scheduled keys are required to be once stored in the register, then should be used for the round function. That is, LEA-256 is not suitable for on-the-fly key generation. The round keys are generated during six clock cycles and stored in registers *t*_2_ to *t*_7_, and are then used for the round function. This requires 9 clock cycles for a round, and 288 clock cycles are needed in all to encrypt a plaintext block.

#### 4.1.8. LEA-256-AREA-2

**Figure 12.**
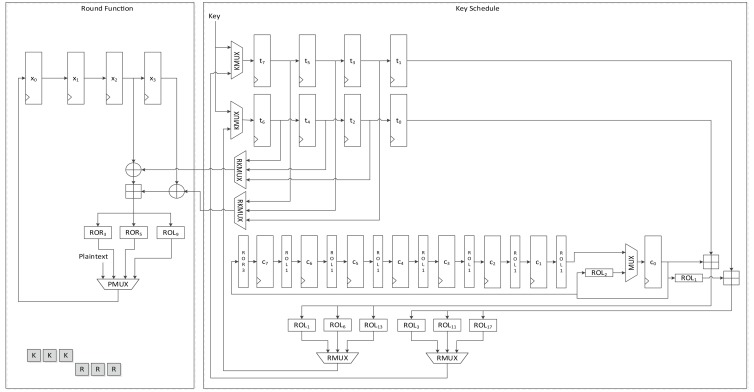
Datapath of LEA-256-AREA-2.

Figure 12 shows another version of area-optimized LEA-256 hardware. This version is similar to LEA-192-AREA-2, which schedules two round keys in a clock. As with LEA-128-AREA-1, on-the-fly round key generation is impossible. Each round key is scheduled once and stored in the register, and is then used for the round function. This reduces the time for scheduling the round key to half of that taken by LEA-256-AREA-1, and it processes one round in 6 clock cycles, thus requiring 192 clock cycles to encrypt a message block.

#### 4.1.9. LEA-256-SPEED

**Figure 13.**
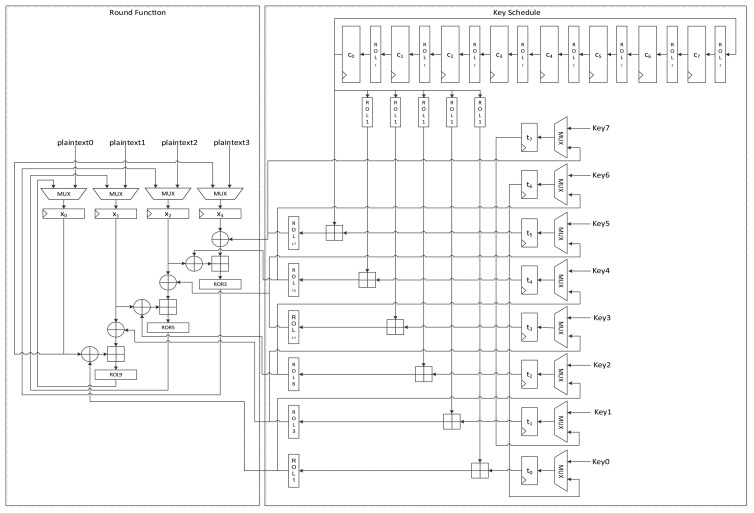
Datapath of LEA-256-SPEED.

The structure of LEA-256-SPEED is depicted in Figure 13. LEA-256 schedules six of eight round keys for a round, and the remaining two and following four keys are used for the next round key generation. Therefore, values in *t*_0_ to *t*_5_ are scheduled and stored in the *t_i_*_+2_ register. The values not used values in *t*_6_ and *t*_7_ are moved to *t*_0_ and *t*_1_, respectively. This implementation requires 32 clock cycles to encrypt a 128-bit plaintext.

## 5. Implementation Results

### 5.1. FPGA

All of the designs described in Section 4 were implemented in Register Transfer Level(RTL) in Verilog. We present the FPGA synthesis result for well-known chips: the Xilinx Virtex 5 series and Altera Cyclone-III series. The Xilinx series was synthesized using ISE 13.4, while the Altera series was synthesized using Quartus-II 11.1sp2.

**Figure 14.**
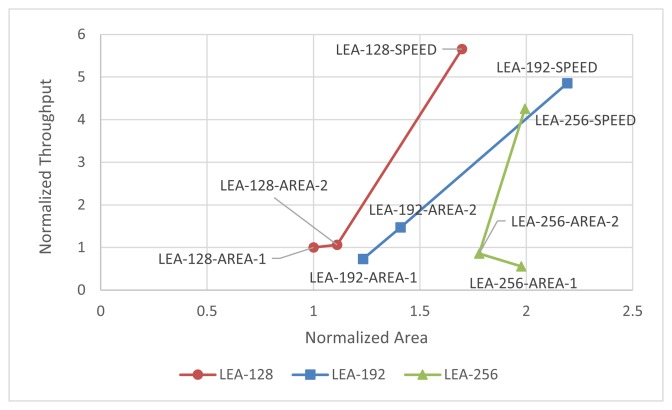
Generalized throughput and area graph to compare relative performance (Xilinx Virtex-5).

The implementation results for the Xilinx Virtex 5 chip are summarized in Table 2. The number of slice elements is counted before being packed into a slice. Looking at the feature, the speed-optimized versions had a higher ATP and throughput per area than the area-optimized versions. This implies that even if replicative XOR and adder logic are reduced in the area-optimized implementation, the amount of reduced logic is negligible. Compared to LEA-128-AREA-1, the size of LEA-128-SPEED is increased by 70%, but the number of cycles is decreased by a factor of 7 times. On the other hand, compared to LEA-128-AREA-1, LEA-128-AREA-2 has a low operating frequency. An analysis of this phenomenon reveals that, in the case of LEA-128-AREA-2, the path from *c*_0_ to *x*_0_ is a critical path, which is the longest path in the implementation. In contrast to LEA-128-AREA-1, LEA-128-AREA-2 has one additional MUX gate in the path from *c*_0_ to *x*_0_, which makes the path longer. On the other hand, LEA-128-AREA-1, LEA-256-AREA-1, and LEA-256-AREA-2, which store the scheduled keys in registers, have short critical paths, since the path from *c*_0_ to *x*_0_ is not required. Consequently, their critical paths are shorter, and the operating frequency is high. Figure 14 shows the normalized throughput and area compared to LEA-128-AREA-1.

**Figure 15.**
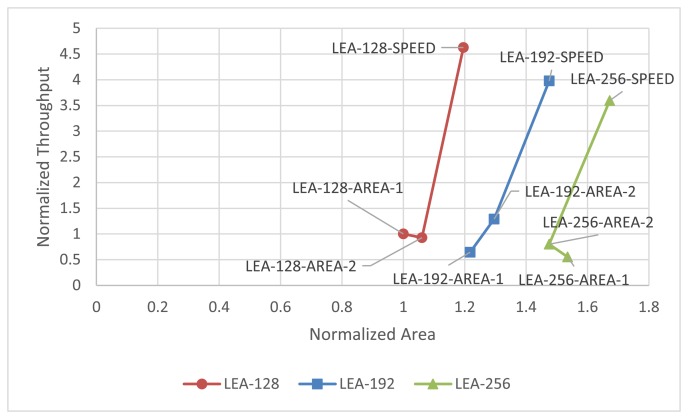
Generalized throughput and area graph to compare relative performance (Altera Cyclone-III).

Table 3 shows the implementation results for Altera Cyclone-III. The overall characteristics of the implementation are similar to those for Xilinx. Also, Figure 15 shows the normalized throughput and size based on LEA-128-AREA-1. The relative implementation results can be found in the figure.

### 5.2. ASIC

We also applied the same RTL code to implement the design into ASIC using Synopsys's Design Compiler B-2008-09.SP5 and the UMC 0.13 µm tech library. The maximum target frequency was 100 MHz, and all the designs met the timing constraints.

Table 4 compares the ASIC implementation results. As in the FPGA implementation case, the speed-opt implementations are not much bigger than the area-opt implementations. The areas of speed-opt versions are increased by about 30%–40%. On the other hand, the throughputs of the speed-opt implementations are much higher than the area-opt ones, resulting in lower ATP and higher throughput per area. Among the same key-length version, there's no significant difference between sequential logic sizes, since requiring the number of flip-flops be alike. However, we can observe that the size of combinational logic is increased in the speed-opt version.

**Figure 16.**
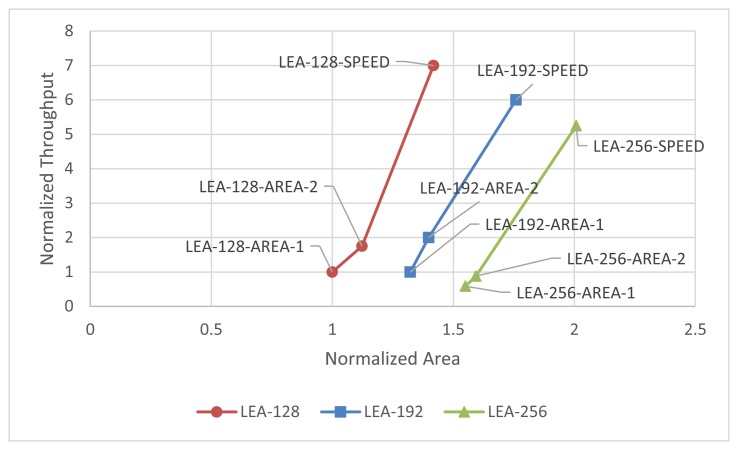
Generalized throughput and area graph to compare relative performance(ASIC).

Figure 16 shows the normalized throughput and size based on LEA-128-AREA-1. The relative implementation results can be found in the figure.

## 6. Comparison

Table 5 compares the ASIC implementation results of LEA with other existing encryption algorithms. First of all, the area of LEA-128-SPEED is larger than other implementations. This is one disadvantage of our implementation. However, the throughput of LEA-128-SPEED is higher than other implementations. This is caused by the low cycles per block. Even though HummingBird2 has smaller cycles per block, the block size of LEA-128-SPEED is much larger. Due to the high throughput, the throughput per area is relatively higher than other implementations except PRESENT and HummingBird2. Although the throughput per area of LEA-128-SPEED is not the best, it shows values similar to PRESENT and HummingBird2, which is known to be efficient. If LEA-128-SPEED is applied to high speed applications, it will be better than both implementations. Even LEA is targeting high software performance, the hardware implementation results are also good compared to other hardware implementations.

## 7. Conclusions

In this paper, we proposed the hardware design and implementation of a new lightweight encryption algorithm, LEA. LEA uses the same round function irrespective of key size. However, there are differences in its method for implementing key scheduling. Based on the key size, we presented suitable hardware designs. For the area-optimized version, we presented a resource-shared structure. Furthermore, by applying on-the-fly key scheduling or scheduling two keys simultaneously, it is possible to reduce the number of clock cycles. For the speed-optimized version, we parallelized all operations required to a round. Due to parallelization, we could achieve high throughput. After presenting the hardware structure of the LEA, we also presented the synthesis result of our design. We implemented our designs into Verilog HDL, then synthesized them to a FPGA chip and ASIC. We targeted commonly-used FPGA chips, and the open-library for ASIC. From the implementation result, we could observe that there is not much area savings of the area-opt version compared to the speed-opt version. This is because the structure of the LEA is too simple, so not much savings can be had by sharing components. Therefore, the speed-opt version shows better throughput per area than the area-opt version, since the area savings of the area-opt version is lower while the speed is significantly lowered. When we compare our implementation result to other results, our result is not the best in throughput per area. However, it does belong to a high position, and it is the best in throughput. We hope our designs can be improved in the future and we present studies on further improvements as future works.

## Figures and Tables

**Table 1.** Notations used to explain LEA algorithm.

**Symbol**	**Meaning**
*P*	128-bit plaintext. *P* = *P*_0_|*P*_1_|*P*_2_|*P*_3_. each *P_n_* is 32-bit.
*C*	128-bit ciphertext. *C* = *C*_0_|*C*_1_|*C*_2_|*C*_3_. each *C_n_* is 32-bit.
*L*(*x*)	Length of bit sequence *x*.
*K*	Master key. *K* = *K*_0_|*K*_1_|…|*K_n_*. *n* = 3 where *L*(*K*) = 128, *n* = 5 where *L*(*K*) = 192, and *n* = 7 where *L*(*K*) = 256.
*X^i^*	Intermediate value of the *i*-th encryption state. $X^{i}=X_{0}^{i}|X_{1}^{i}|X_{2}^{i}|X_{3}^{i}$ where 0 ≤ *i* < *r*. Each $X_{n}^{i}$ is 32-bit.
*T^i^*	Intermediate value of the *i*-th key schedule state. $T^{i}=T_{0}^{i}|T_{1}^{i}|T_{2}^{i}|T_{3}^{i}$ where 0 ≤ *i* < *r*. Each $T_{n}^{i}$ is 32-bit.
*δ*_0_, *δ*_1_,*…*, *δ_n_*	Constant value used for the key schedule. *n* = 3 where *L*(*K*) = 128, *n* = 5 where *L*(*K*) = 192, and *n* = 7 where *L*(*K*) = 256.
*r*	Number of round iterations. *r* = 24 where *L*(*K*) = 128, *r* = 28 where *L*(*K*) = 192, and *r* = 32 where *L*(*K*) = 256.
*RK^i^*	192-bit round key used for the i-th round. $RK^{i}=RK_{0}^{i}|RK_{1}^{i}|RK_{2}^{i}|RK_{3}^{i}|RK_{4}^{i}|RK_{5}^{i}$ where 0 ≤ *i* < *r*. Each $RK_{n}^{i}$ is 32-bit.
⊕	XOR operation.
⊞	Addition modulo 2^32^.
*ROL_i_*(*x*)	*x*-bit left rotation.
*ROR*_i_(*x*)	*x*-bit right rotation.

**Table 2.** Comparison of implementation results using Xilinx Virtex 5.

**Designs**	**Cycles**	**Max. Freq.**	**Latency @ max freq (µs)**	**Latency @ 10MHz (µs)**	**Throughput (Mbps)**	**Area (Slice Element)**			**ATP**	**Throughput/Area**
						**Reg**	**LUT**	**Total**		
LEA-128-AREA-1	168	269.658	0.62	16.8	205.45	392	249	503	311.86	0.41
LEA-128-AREA-2	96	163.861	0.59	9.6	218.48	388	306	559	329.81	0.39
LEA-128-SPEED	24	217.806	0.11	2.4	1,161.63	386	713	854	93.94	1.36
LEA-192-AREA-1	168	197.797	0.85	16.8	226.05	423	408	620	527	0.36
LEA-192-AREA-2	84	198.364	0.42	8.4	453.40	514	403	709	297.78	0.64
LEA-192-SPEED	28	218.250	0.13	2.8	1,496.57	508	911	1,103	143.39	1.36
LEA-256-AREA-1	288	257.652	1.12	28.8	229.02	663	713	994	1,113.28	0.23
LEA-256-AREA-2	192	169.2	1.13	19.2	225.60	649	987	1,003	1,133.4	0.22
LEA-256-SPEED	32	126.23	0.25	3.2	1,009.84	645	1,131	1,137	284.3	0.89

**Table 3.** Comparison of implementation results using Altera Cyclone-III.

**Designs**	**Cycles**	**Max. Freq.**	**Latency @ max Freq (µs)**	**Latency @ 10MHz (µs)**	**Throughput (Mbps)**	**Area (Slice)**			**ATP**	**Throughput/Area**
						**Reg**	**Comb**	**Total LE**		
LEA-128-AREA-1	168	184.47	0.91	16.8	140.55	392	632	680	618.8	0.21
LEA-128-AREA-2	96	97.98	0.98	9.6	130.64	391	721	721	706.6	0.18
LEA-128-SPEED	24	121.91	0.20	2.4	650.19	389	812	813	162.6	0.80
LEA-192-AREA-1	168	119.03	1.41	16.8	136.03	520	823	828	1,167.5	0.16
LEA-192-AREA-2	84	119.13	0.71	8.4	272.30	519	864	881	625.5	0.31
LEA-192-SPEED	28	122.35	0.23	2.8	838.97	517	1,003	1,003	230.7	0.84
LEA-256-AREA-1	288	174.76	1.65	28.8	155.34	650	996	1,044	1,722.6	0.15
LEA-256-AREA-2	192	169.2	1.13	19.2	225.60	649	987	1,003	1,133.4	0.22
LEA-256-SPEED	32	126.23	0.25	3.2	1,009.84 645		1,131	1,137	284.3	0.89

**Table 4.** Comparison of ASIC implementation results. (UMC 0.13 um, Target frequency: 100 MHz).

**Designs**	**Cycles**	**Latency @100MHz (µs)**	**Throughput (Mbps)**	**Area(GE)**			**ATP**	**Throughput/Area**
				**Comb.**	**Seq.**	**Total**		
LEA-128-AREA-1	168	1.68	76.19	1,707.5	2,118.5	3,826	6,427.7	0.02
LEA-128-AREA-2	96	0.96	133.33	2,157.75	2,137.75	4,295.5	4,123.7	0.03
LEA-128-SPEED	24	0.24	533.33	3,309.25	2,116.75	5,426	1,302.2	0.10
LEA-192-AREA-1	168	1.68	114.29	2,245	2,813.5	5,058.5	8,498.3	0.02
LEA-192-AREA-2	84	0.84	228.57	2,538.5	2,812.5	5,351	4,494.8	0.04
LEA-192-SPEED	28	0.28	685.71	3,907.75	2,823.5	6,731.25	1,884.8	0.10
LEA-256-AREA-1	288	2.88	88.89	2,376.5	3,555.75	5,932.25	17,084.9	0.01
LEA-256-AREA-2	192	1.92	133.33	2,440.75	3,655.5	6,096.25	11,704.8	0.02
LEA-256-SPEED	32	0.32	800.00	4,142.5	3,540	7,682.5	2,458.4	0.10

**Table 5.** Comparison to other encryption algorithms.

**Algorithms**	**Key Length**	**Block Size**	**Cycles/Block**	**Throughput @100KHz (Kbps)**	**Tech. (µm)**	**Area (GE)**	**Throughput/Area**
DESL [11]	56	64	144	44.4	0.18	1,848	0.024026
KATAN [4]	80	64	255	25.1	0.13	1,054	0.023814
HIGHT [2]	128	64	34	188.2	0.25	3,048	0.061745
PRESENT [3]	128	64	32	200.0	0.18	1,570	0.127389
PRESENT [8]	128	64	547	11.7	0.18	1,075	0.010884
HummingBird2 [5]	128	16	4	400.0	0.18	3,220	0.124224
HummingBird2 [5]	128	16	20	80.0	0.18	2,159	0.037054
AES [7]	128	128	226	56.6	0.13	2,400	0.023583
LED [6]	128	64	1,872	3.4	0.18	1,265	0.002688
LEA-128-SPEED	128	128	24	533.3	0.13	5,426	0.098286
DESXL [11]	184	64	144	44.4	0.18	2,168	0.02048
LEA-196-SPEED	196	128	28	457.1	0.13	6,731	0.06791
LEA-256-SPEED	256	128	32	400.0	0.13	7,683	0.052063
